# Silver/Polypyrrole-Functionalized Polyurethane Foam Embedded Phase Change Materials for Thermal Energy Harvesting

**DOI:** 10.3390/nano11113011

**Published:** 2021-11-09

**Authors:** Dongli Fan, Yuan Meng, Yuzhuo Jiang, Siyi Qian, Jie Liu, Yuzhi Xu, Dangsheng Xiong, Yufeng Cao

**Affiliations:** 1 School of Materials Science & Engineering, Nanjing University of Science and Technology, Nanjing 210094, China; fdlsky@ntu.edu.cn; 2 School of Chemistry and Chemical Engineering, Nantong University, Nantong 226019, China; meng971123@163.com (Y.M.); yuzhuojiang@163.com (Y.J.); 18751310311@163.com (S.Q.); jliu93@ntu.edu.cn (J.L.); 3 Institute of Chemical Industry of Forest Products, Chinese Academy of Forestry, Nanjing 210042, China; xuyuzz@163.com

**Keywords:** phase change materials, silver/polypyrrole-functionalized polyurethane foam, solar–thermal energy conversion

## Abstract

Conversion of solar energy into thermal energy stored in phase change materials (PCMs) can effectively relieve the energy dilemma and improve energy utilization efficiency. However, facile fabrication of form-stable PCMs (FSPCMs) to achieve simultaneously energetic solar–thermal, conversion and storage remains a formidable challenge. Herein, we report a desirable solar–thermal energy conversion and storage system that utilizes paraffin (PW) as energy-storage units, the silver/polypyrrole-functionalized polyurethane (PU) foam as the cage and energy conversion platform to restrain the fluidity of the melting paraffin and achieve high solar–thermal energy conversion efficiency (93.7%) simultaneously. The obtained FSPCMs possess high thermal energy storage density (187.4 J/g) and an excellent leak-proof property. In addition, 200 accelerated solar–thermal energy conversion-cycling tests demonstrated that the resultant FSPCMs had excellent cycling durability and reversible solar–thermal energy conversion ability, which offered a potential possibility in the field of solar energy utilization technology.

## 1. Introduction

The growing public concerns about energy shortage and environmental degradation caused by the over-consumption of non-renewable energy resources have encouraged people to explore and develop available renewable energy and energy conversion techniques for the sustainable development of human civilization [1,2,3]. Solar energy is a promising renewable energy for human beings and the earth’s surface receives a large quantity of solar irradiation every day. The conversion of solar energy into heat is an appealing way to alleviate the energy crisis and environmental concerns and improve solar energy utilization efficiency [4,5,6,7]. However, solar energy is a representative time-dependent energy resource with an intermittent and discontinuous attribute, which has undermined its widespread exploitation and commercial applications. Latent heat storage technologies based on organic PCMs can gather thermal energy from solar irradiation, which can conquer the intermittency and instability of solar energy [8,9,10]. In this process, solar energy is captured and converted into thermal energy stored within the organic PCMs via solid–liquid phase change. When solar irradiation is not available, the stored latent heat can be released to regulate the local temperature fluctuation.

Over the past few decades, organic PCMs have been extensively used in various high technology fields, including solar energy conversion and storage [11,12,13], smart thermos-regulated textiles [14,15,16], thermal protection electronic devices [17,18] and healthcare services [19,20,21] due to its high latent heat storage capacity, desired physicochemical stability and remarkable energy-saving capability [22,23]. Nevertheless, the possibility of liquid leakage and inferior solar–thermal energy conversion efficiency has severely restricted the practical applications for most commonly available organic PCMs. One scalable method is to design and develop form–stable PCMs (FSPCMs) with improved solar–thermal energy conversion ability, which can not only remove the leakage threats but also gather thermal energy from solar irradiation [24,25,26]. Polyaniline (PANI) [27,28], polydopamine (PDA) [29,30,31] and polypyrrole (PPy) [32,33] as kinds of polymer-based light absorber materials have been introduced into organic PCMs due to their impressive solar–thermal conversion property. Nevertheless, stable solar–thermal energy harvesting and high latent heat energy storage density with ignorable heat waste for the FSPCMs remains one of the hotspots due to liquid leakage, low heat–conducting property and limited energy conversion efficiency, obstructing their further development and business application.

Herein, we constructed a series of novel FSPCMs with impressive solar–thermal energy conversion ability, where the silver/polypyrrole composites coating polyurethane (PU) foam was used as the building scaffolds and energy conversion platform and paraffin as thermal energy storage mediums (see Figure S1). The 3D continuous porous structures allowed the PU foam to be viewed as the building scaffolds to capture and constrain the melting paraffin, promising the FSPCMs an outstanding anti-leakage property. The coated silver/polypyrrole composites on PU skeletons essentially provided an impressive solar–thermal energy conversion ability and heat–conducting property, thus collecting solar irradiation and converting it into thermal energy, which was delivered along with the skeletons of the PU foam and stored in PCMs. The preparation, characterization and application of the developed FSPCMs were systematically investigated with the aim of obtaining positively improved FSPCMs for potential applications, satisfying the needs of different solar–thermal energy conversion and storage systems.

## 2. Experimental Section

### 2.1. Materials

Pyrrole (99%), Fe(NO_3_)_3_ (≥98.5%) and ammonium persulfate (APS, ≥98.5%) were obtained from Nantong Feiyu Biochemical Co., Ltd., Nantong, China. AgNO_3_ (≥99.8%) and paraffin (PW, *T*_m_ ≈ 56–58 °C) was provided by Sino Pharm Chemical Reagent Co., Ltd., Beijing, China. Deionized water was obtained from Chinese Local Supermarkets. Polyurethane (PU) foam was prepared according to a previous literature procedure with some modifications [34]. All chemicals were utilized as received without further purification.

### 2.2. Preparation of PPy@PU

The PPy@PU was fabricated according to a previous literature procedure with a minor modification [35]. Generally, a piece of PU foam with a thickness of 15 mm was immersed into 0.1 M pyrrole aqueous solution at room temperature for 24 h to guarantee penetration equilibrium of pyrrole into the PU 3D network structures. Subsequently, the abovementioned PU with pyrrole infiltrative was immediately immersed into 0.1 M APS aqueous solution for 1 h, where the color of the PU changed from light yellow to black. Finally, the as-fabricated PPy@PU was repeatedly washed with deionized water, followed by freeze-drying. The prepared PPy@PU was denoted as UP.

### 2.3. Preparation of Ag/PPy@PU

A block of PU foam with a thickness of 15 mm was immersed into 0.002 M pyrrole aqueous solution at environment temperature for 24 h to guarantee penetration equilibrium of pyrrole into the PU 3D porous structures. Subsequently, the abovementioned PU with pyrrole infiltrative was immediately immersed into an oxidant aqueous solution for 1 week to complete the polymerization, where the color of the solution changed from light yellow to black. Finally, the as-prepared Ag/PPy@PU was thoroughly washed with deionized water, followed by freeze–drying. The aqueous solution of AgNO_3_ and Fe(NO_3_)_3_ was employed as the oxidant, and the mole ratio of oxidants/pyrrole was 2.5. The concentration of the oxidants used in the polymerization is shown in Table S1. The fabricated Ag/PPy@PU was denoted as UPA*x* (*x* = 1, 2, 3 and 4, which represents the concentration of AgNO_3_ in oxidants aqueous solution).

### 2.4. Preparation of the FSPCMs

The as-prepared UPA*x* were immersed into the melted paraffin to guarantee penetration equilibrium of the melted paraffin into the 3D porous structures of UPA*x* via vacuum-assisted conditions and was maintained at 80 °C for 6 h; afterwards, the FSPCMs were obtained and referred to as UPA*x*/PW. The fabrication process for the UP/PW was the same.

### 2.5. Characterizations

The prepared samples’ morphologies were observed using SEM observation (ZEISS Gemini SEM 300, Baden-Württemberg, Germany). FT-IR spectra (Nicolet Nexus 6700, Madison, WI, USA) and XRD analysis (Bruker D8 advance, Karlsruhe, Germany) were conducted to investigate the structure and crystalline property of the prepared samples in this work, respectively. The melting and crystallization enthalpies of samples were measured via a DSC analysis (DSC 2500, TA Instruments, New Castle, PA, USA). The thermal conductivity of samples was confirmed by a transient plane heat source (hot disk) method (DRE-III, Xiangtan Xiangyi Instrument Co., Ltd., Xiangtan, China). On the other hand, a solar simulator (CEL-PF300-T10, Beijing China Education Au-light Co., Ltd., Beijing, China) and an infrared camera (FLIR E5-XT, North Billerica, MA, USA) were employed to observe the solar–thermal energy conversion, storage and release behavior of the prepared samples. Meanwhile, the solar–thermal energy harvesting and storage of sample during 200 times cycling tests was confirmed by simulative light source and DSC analysis, and the structural stability was also verified through FT-IR and XRD analysis, respectively.

## 3. Results and Discussion

### 3.1. Morphology and Structure of the FSPCMs

**Figure 1:**
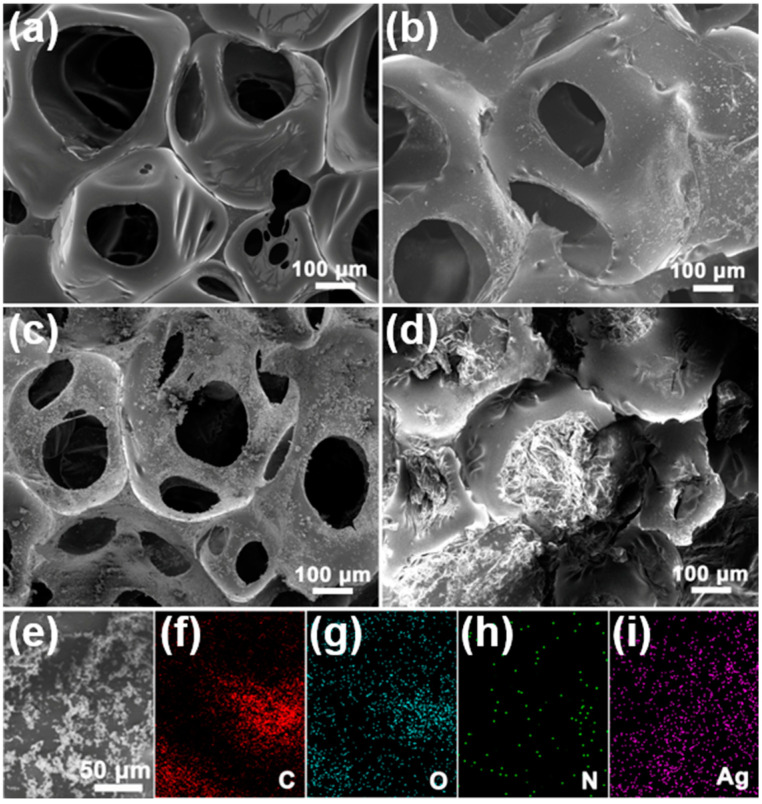
SEM images of (**a**) PU, (**b**) UP, (**c**) UPA3, (**d**) UPA3/PW and (**e**–**i**) elemental mapping images of UPA3.

SEM images, as shown in Figure 1a–c, were utilized to monitor the morphologies evolution of PU, UP, UPA*x* and UPA*x*/PW. The as-prepared PU foam showed a 3D continuous porous structure, and the walls of pores are relatively smooth (Figure 1a). The UP skeletons were also observed as presented in Figure 1b, and the walls of UP skeletons exhibited a coarse landscape texture due to the pyrrole in situ polymerization on the surface of PU skeletons. Taking UPA3 as an example, after further modifying the PU with Ag/PPy composites, the walls of its skeletons became rougher (Figure 1c), which was more conducive to arresting and constraining the melting PW due to capillary effect and intermolecular interaction. To further confirm the distribution of Ag/PPy composites within the PU skeletons, the elemental mapping measurement was conducted, and the corresponding results are presented in Figure 1e–i. As we can see, the elemental C, O, N and Ag mainly assembled on the walls of PU skeletons homogeneously. Moreover, thanks to the local surface plasma resonance effect (LSPR) of silver [36], the UPA3 had enhanced solar harvesting ability and heat conductivity, resulting in the rapid temperature increase of 98.7 °C of the UPA3 in 1.0 min under 300 mW/cm^2^ irradiation power (Figure S2a); in contrast, the pure PU foam only reached 49.7 °C (Figure S2b). The UPA*x* were immersed into the melting paraffin to prepare a group of novel FSPCMs under the vacuum-assisted condition. The morphology of the representative UPA3/PW was investigated through SEM observation. Figure 1d showed that the absorbed PW fully filled in the inner cavities or tightly intertwined with the skeletons of the UPA3, indicating the UPA*x* offered a 3D continuous porous platform to effectively arrest and constrain the melting PW molecules. On the other hand, as shown in Figure S3, even if the working temperature was above PW’s phase-transition temperature, the FSPCMs not only maintained a solid state but also were leak-proof.

**Figure 2:**
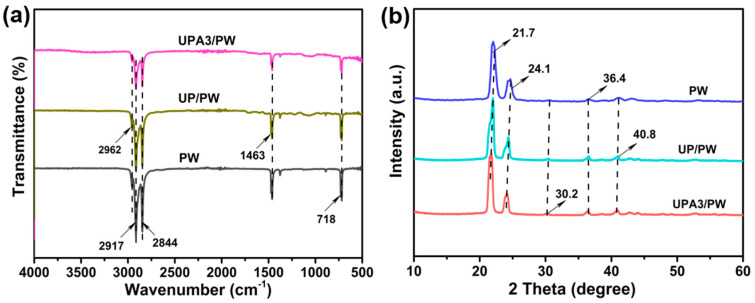
(**a**) FT–IR spectra and (**b**) XRD patterns of PW, UP/PW and UPA3/PW.

The structural feature and crystallization property of the developed FSPCMs were investigated by FT–IR spectra and XRD diffraction, and the results are depicted in Figure 2 and Figure S4. As we can see, no new characteristic absorption bands and diffraction peaks were evidently observed, demonstrating only that physical interaction existed between the components of the FSPCMs, and the crystalline property of PW was also not be destroyed by the volume restriction effect of the UPA*x*.

### 3.2. Thermal Properties of the FSPCMs

**Figure 3:**
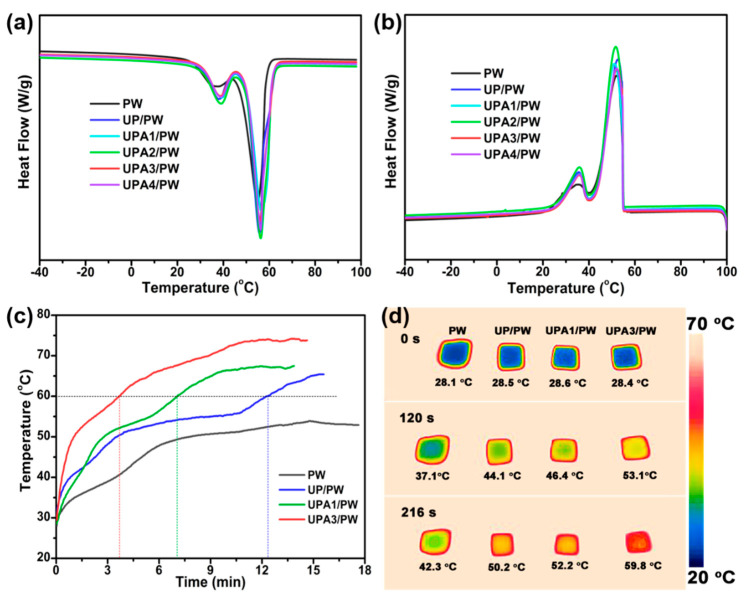
Thermal properties of PCCs. The DSC (**a**) heating and (**b**) cooling curves of PW and PCCs; (**c**) Temperature–time curves and (**d**) Infrared thermal images of PW, UP/PW, UPA1/PW and UPA3/PW during the charging process.

Latent heat storage density is a critical parameter in determining the development prospect of FSPCMs in practical applications. DSC analysis was used to measure the melting and crystallization enthalpies of the prepared FSPCMs in this work, and the experimental results are presented in Figure 3a,b. As depicted in Figure 3a,b, a melting endothermic peak and a crystallization exothermic peak appeared at 54.6 °C and 52.2 °C on the DSC heating and cooling curves of pristine PW, corresponding to the thermal energy charging and discharging process, respectively. The melting temperatures of UP/PW, UPA1/PW, UPA2/PW, UPA3/PW and UPA4/PW changed by 0.9, 1.8, 1.5, 1.9 and 1.5 °C, and the freezing temperatures changed by 0.5, −1.0, −0.4, −1.0, and 0.1 °C, respectively, which was slightly different than that of pure PW. In part, this is because the steric effect of the PU foam imposed restrictions on the molecular movement and spatial arrangement of PW and postponed the phase transformation behavior [37]. It can be seen from Table S2 that the obtained FSPCMs offered a relatively high latent heat storage density, and the melting and crystal enthalpies ranged from 185.2 to 197.9 J/g and 182.0 to 196.7 J/g, respectively. The available latent heat storage capacity of the FSPCMs was less than that of pristine PW, because the PU foam in the FSPCMs cannot offer up the melting and crystal enthalpies. In addition, the melting and crystallization properties of PW in the FSPCMs were blocked at some level by the PU foam, which also led to the partial decline of the melting and crystal enthalpies. Nevertheless, as shown in Table S3, the latent heat storage density of the developed FSPCMs in this work reached 87.1 to 93.2% of pristine PW, which was higher or comparable to the recently reported data [27,28,38,39,40,41,42,43].

In addition, it can be seen from Figure S5 that the pure PW showed a low thermal conductivity of 0.271 W m^−1^ K^−1^, whereas the thermal conductivities of PU/PW UP/PW and UPA3/PW sequentially increased. The maximum thermal conductivity is up to 0.391 W m^−1^ K^−1^ for the UPA3/PW, which is 144% higher than that of the pure PW, which could contribute to the fast charging and discharging. The heat transfer rate of samples during the charging process was also investigated and shown in Figure 3c,d. As presented in Figure 3c, to achieve a temperature of 60 °C from an ambient temperature, the heating times of UP/PW, UPA1/PW and UPA3/PW were 12.3, 7.0 and 3.7 min, respectively. It was difficult to reach 60 °C for pure paraffin due to its inferior heat-conducting property. Furthermore, as shown in Figure 3d, the heating rate of the UPA3/PW was evidently faster than other samples under the same interval time. The above results suggest that the heat transfer rate of these FSPCMs increased with the increasing doping amount of Ag/PPy composites, which makes them a promising opportunity for rapid thermal energy storage.

### 3.3. Solar–Thermal Energy Harvesting and Storage

**Figure 4:**
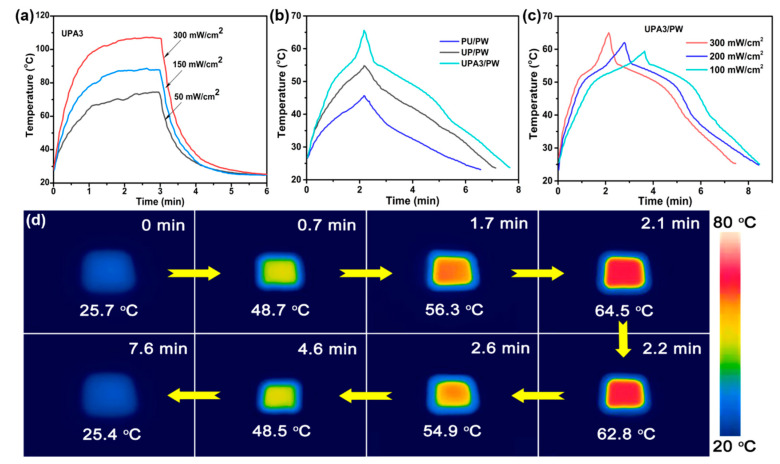
Time–temperature curves of (**a**) UPA3, (**b**) PU/PW, UP/PW and UPA3/PW, and (**c**) UPA3/PW (13 × 13 × 3.0 mm, mass: 142.9 mg) under various solar intensity; (**d**) Infrared thermal images showing the solar–thermal energy conversion, storage and release behavior of the UPA3/PW.

High energy conversion efficiency and fast thermal transmission are essential to the FSPCMs. PPy as an efficient solar–thermal agent has been successfully used to improve the solar absorption capability of the organic PCMs [32,33], but its thermal conductivity was relatively poor. Silver nanoparticles could enhance the heat-conducting property and improve the visible light absorption for the organic PCMs simultaneously [36]. Therefore, in these developed FSPCMs, the assembled Ag/PPy composites promised satisfactory solar–thermal energy conversion ability and the PU foam coating with Ag/PPy composites contributed to the avenues for rapid energy transfer so that the absorbed thermal energy could be charged into the PW. Figure 4a shows that the empty UPA3 was irradiated under 50~300 mW/cm^2^ light power. The temperature of the UPA3 rapidly rose with the increase in light power and ultimately reached an equilibrium temperature (*T*_e_), where the absorbed heat from solar irradiation balanced the heat loss from the UPA3 with the surroundings. When the solar irradiation was not available, the temperature of the sample fell to room temperature in short order, showing the rapid thermal runaway. Therefore, the result indicates that the UPA3 offers desirable light–thermal energy conversion ability and excellent thermal transmission performance. For the obtained FSPCMs, the introduced PPy and Ag/PPy composites can effectively capture and convert light into thermal energy stored by PW through the phase-transition process. As shown in Figure 4b, the heating rate of the UPA3/PW was evidently faster than PU/PW and UP/PW at the same irradiation time, indicating that the Ag/PPy composites offer higher solar harvesting and conversion efficiency than pure PPy. On the other hand, we can see from Figure 4c that the temperature–time evolution curves of the UPA3/PW under different light power revealed obvious endothermic and exothermic platforms, corresponding to the heat energy storage and release, respectively. The solar–thermal energy conversion efficiency (η) of the composite FSPCMs was calculated according to the Equation [6]:

(1) $$\eta =\frac{m\;\times \;\Delta H_{m}}{IA\;\times \;\left(t_{f}{\;-\;t}_{0}\right)}\;\times \;100\%$$

where *m* is the mass of the composite FSPCMs, Δ*H*_m_ is the melting enthalpy of the composite FSPCMs, *I* is the irradiation intensity, *A* is the surface area of the sample and *t_f_* and *t*_0_ are the termination and onset time of phase transformation, respectively. Therefore, the calculated η value of the UPA3/PW reached 93.7% at 300 mW/cm^2^ power intensity, which is higher than some other reported data (60%~86%) [32,44,45,46,47]. In addition, an infrared camera recorded the temperature changes of the UPA3/PW under 300 mW/cm^2^ light power. It can be seen from Figure 4d that the temperature rapidly increased from 25.7 to 48.7 °C in 0.7 min as the UPA3/PW absorbed heat energy directly from solar. Subsequently, the temperature was maintained between 48.7 and 56.3 °C for about 1 min, reaching the melting platform (Figure 3c), where the harvested heat was directly stored into the PW through the phase-transformation process. As irradiation time passed by, the temperature ultimately reached a saturation temperature (64.5 °C) in 2.1 min. On the other hand, once the light irradiation was not available, the temperature of the specimen declined quickly until it reached an obvious freezing platform between 54.9 and 48.5 °C, demonstrating that latent heat stored within PW can be discharged via natural cooling. This exothermic process was maintained for about 2.0 min, which contributed to the temperature regulation for smart fabrics, electron devices and so on. The results prove that the developed UPA3/PW could collect available thermal energy from solar irradiation and offers potential use in the field of solar energy utilization technique.

### 3.4. Cycling Durability of the FSPCMs

**Figure 5:**
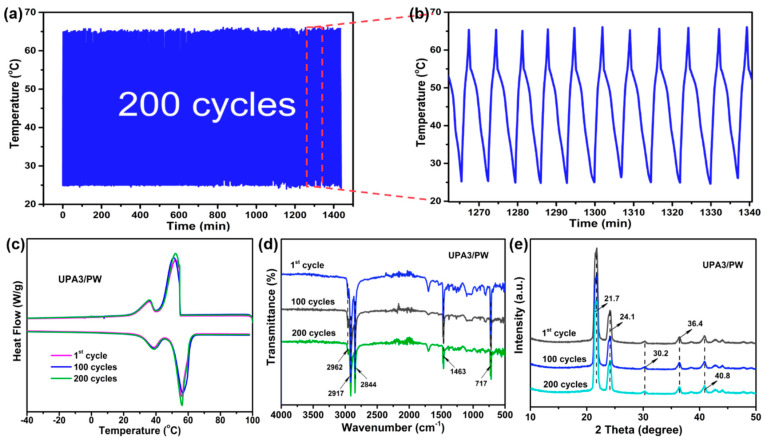
(**a**,**b**) Time–temperature evolution curves during 200 accelerated solar–thermal energy conversion cycling tests; (**c**) DSC curves, (**d**) FT–IR spectrum and (**e**) XRD patterns of the UPA3/PW before and after cycling tests.

The results of the accelerated energy conversion cycling tests evaluating the cycling durability of the UPA3/PW are shown in Figure 5. When the illumination intensity was 300 mW/cm^2^ light power, the UPA3/PW reached a saturation temperature (*T*_s_ = 64.5 °C) within 2.1 min, which declined as the solar simulator was evacuated (Figure 4c). In addition, we can see from Figure 5a,b that the time–temperature changing curves of the UPA3/PW during the 200 accelerated solar–thermal energy conversion cycling tests remained virtually unchanged. The heat storage properties of the UPA3/PW show almost no fluctuation before and after the accelerated cycling tests (Figure 5c and Table S2). Moreover, as shown in Figure 5d,e, the FT–IR spectra and XRD patterns of the UPA3/PW did not detect new absorption peaks before and after 200 accelerated solar–thermal energy conversion cycling tests either, showing that the accelerated cycling tests had not exerted a negative impact on its structure and crystallizing behavior. The above results show that the UPA3/PW offered excellent cycling durability, unlocking a potential possibility to use it in commercial applications.

## 4. Conclusions

In this work, we firstly fabricated a series of silver/polypyrrole composites coating PU foam (UPA*x*) with an impressive light absorption ability and 3D multi-porous structure. Subsequently, we designed and constructed a novel solar–thermal energy harvesting, storage and release system, in which the UPA*x* served as building skeletons and energy conversion platform simultaneously, and PW served as latent heat storage materials. The resultant FSPCMs not only had an acceptable leak-proof performance, but also a latent heat energy storage density as high as 187.4 J/g. Meanwhile, silver/polypyrrole composites, a commendable solar absorption agent, endowed the composite FSPCMs with excellent solar–thermal energy conversion efficiency (93.7%). In addition, 200 accelerated solar–thermal energy conversion-cycling tests proved that the prepared FSPCMs in this work provided stable and reversible solar–thermal energy conversion property, which unlocked a potential possibility in the utilization of solar energy.

## Data Availability

The data presented in this study are available on request from the corresponding author.

## Supplementary Materials

The following are available online at https://www.mdpi.com/article/10.3390/nano11113011/s1, Figure S1: Schematic description of the formation of the FSPCMs (UPA*x*/PW); Figure S2: Infrared thermal image of (a) UPA3 and (b) pure PU foam under 300 mW/cm^2^ in 1.5 min; Figure S3: Shape stability of paraffin and the FSPCMs; Figure S4: (a) FT-IR spectra and (b) XRD patterns of PU foam and PPy; Figure S5: The thermal conductivity of PW, PU/PW, UP/PW and UPA3/PW; Table S1: Concentrations of AgNO_3_ and Fe (NO_3_)_3_ used for the oxidation of pyrrole; Table S2: DSC data of the prepared FSPCMs in this work; Table S3: Comparison of the thermal properties of the FSPCMs with other reported paraffin-based PCMs.
