# Supplementary material for: Silver/Polypyrrole-Functionalized Polyurethane Foam Embedded Phase Change Materials for Thermal Energy Harvesting
Source: Nanomaterials (Basel). 2021 Nov 9;11(11):3011. doi: 10.3390/nano11113011 (PMC8621923; doi:10.3390/nano11113011)
Supplement: Supplementary file 1 [file nanomaterials-11-03011-s001.zip › nanomaterials-1421668-supplementary.pdf]

nanomaterials

Supplementary Materials

Silver/Polypyrrole-Functionalized Polyurethane Foam Embedded Phase Change Materials for Thermal Energy Harvesting

Dongli Fan [1,2], Yuan Meng [2], Yuzhuo Jiang [2], Siyi Qian [2], Jie Liu [2], Yuzhi Xu [3], Dangsheng Xiong [1,*] and Yufeng Cao [2,*]

1 School of Materials Science & Engineering, Nanjing University of Science and Technology, Nanjing 210094, China; fdlsky@ntu.edu.cn
2 School of Chemistry and Chemical Engineering, Nantong University, Nantong 226019, China; meng971123@163.com (Y.M.); yuzhuojiang@163.com (Y.J.); 18751310311@163.com (S.Q.); jliu93@ntu.edu.cn (J.L.)
3 Institute of Chemical Industry of Forest Products, Chinese Academy of Forestry, Nanjing 210042, China; xuyuzz@163.com
* Correspondence: xiongds@163.com (D.X.); yufengntu@ntu.edu.cn (Y.C.)

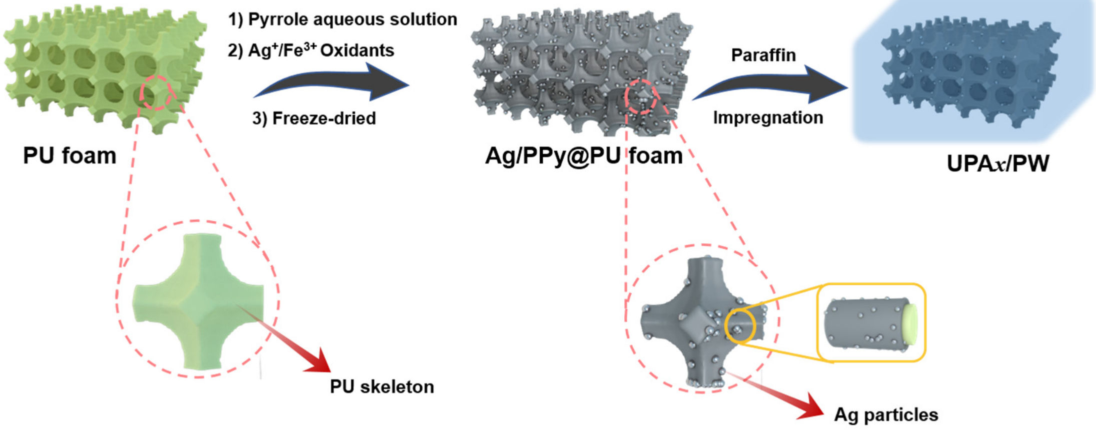

Figure S1. Schematic description of the formation of the FSPCMs (UPA*x*/PW).

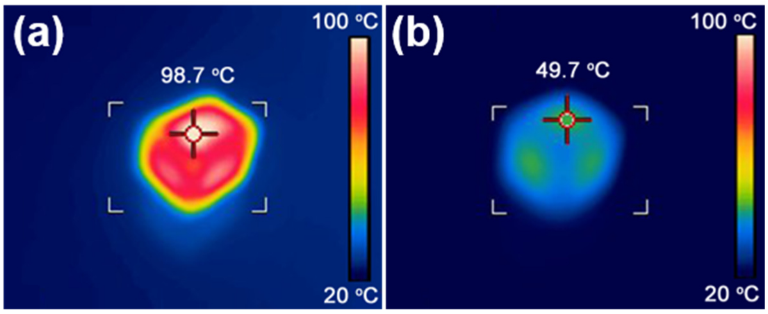

Figure S2. Infrared thermal image of (**a**) UPA3 and (**b**) pure PU foam under 300 mW/cm² in 1.0 min.

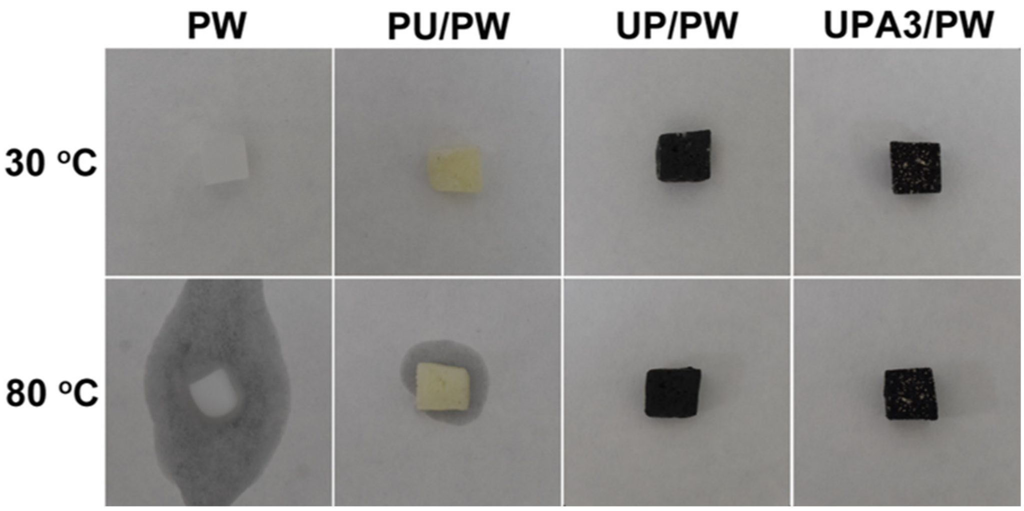

Figure S3. Shape stability of paraffin and the FSPCMs.

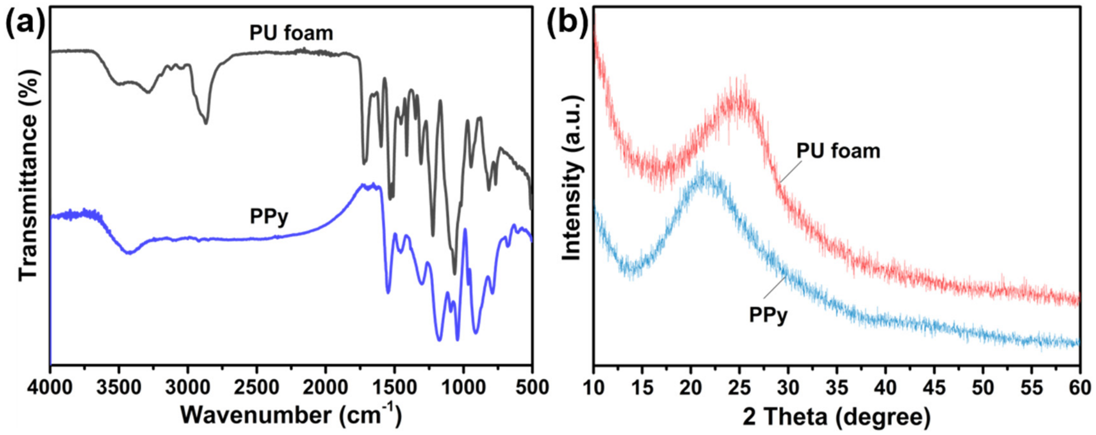

Figure S4. (**a**) FT-IR spectra and (**b**) XRD patterns of PU foam and PPy.

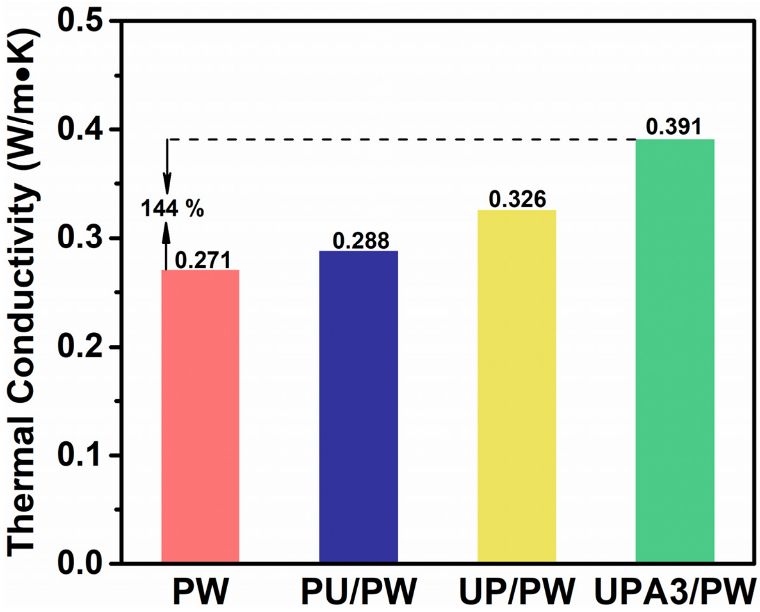

Figure S5. The thermal conductivity of PW, PU/PW, UP/PW and UPA3/PW.

Table S1. Concentrations of $AgNO_3$ and Fe $(NO_3)_3$ used for the oxidation of pyrrole.

Samples	$AgNO_3$ (mmol/L)	$Fe(NO_3)_3$ (mmol/L)	Mass Fraction of $AgNO_3$ (%)
UP	0	2.5	0
UPA1	1.0	1.5	40
UPA2	1.5	1.0	60
UPA3	2.0	0.5	80
UPA4	2.5	0	100

Table S2. DSC data of the prepared FSPCMs in this work.

Samples	Melting Process		Freezing Process	
	T_m (°C)	ΔH_m (J/g)	T_c (°C)	ΔH_c (J/g)
PW	54.8	212.4	52.0	211.8
UP/PW	55.7	191.9	52.5	190.0
UPA1/PW	56.6	193.6	51.0	190.9
UPA2/PW	56.3	197.9	51.6	196.7
UPA3/PW	56.7	186.9	51.0	187.0
UPA3 after 100 cycles	56.2	186.5	52.1	188.7
UPA3 after 200 cycles	56.1	187.4	52.2	186.2
UPA4/PW	56.3	185.2	52.1	182.0

Table S3. Comparison of the thermal properties of the FSPCMs with other reported PW-based PCMs.

Samples	T_m (°C)	ΔH_m (J/g)	ξ (%) [a]	References
PW/PANI	55.6	166.2/-	99.0	[27, 28]
PANI@cobalt-PW	63.1	145.96	95.2	[38]
Paraffin/CAN70	52.4	142.1	63.8	[39]
CPCM#1	46.51	150	88.2	[40]
Pa@SiO_2	43.39	191.2	76.1	[41]
MF-PW30	56.8	139.8	89.8	[42]
EP/P50/GP50	61.2	112.7	50.6	[43]
UPA3/PW	56.1	187.4	88.2	This work

[a] ξ refers to the percentage of paraffin in corresponding samples using the equation $\xi = (\Delta H_m/\Delta H_{pure\ paraffin})$ *100 %.
